# Supplementary material for: Excavating hidden adsorption sites in metal-organic frameworks using rational defect engineering
Source: Nat Commun. 2017 Nov 16;8:1539. doi: 10.1038/s41467-017-01478-4 (PMC5691151; doi:10.1038/s41467-017-01478-4)
Supplement: Supplementary file 1 — Supplementary Information [file 41467_2017_1478_MOESM1_ESM.docx]

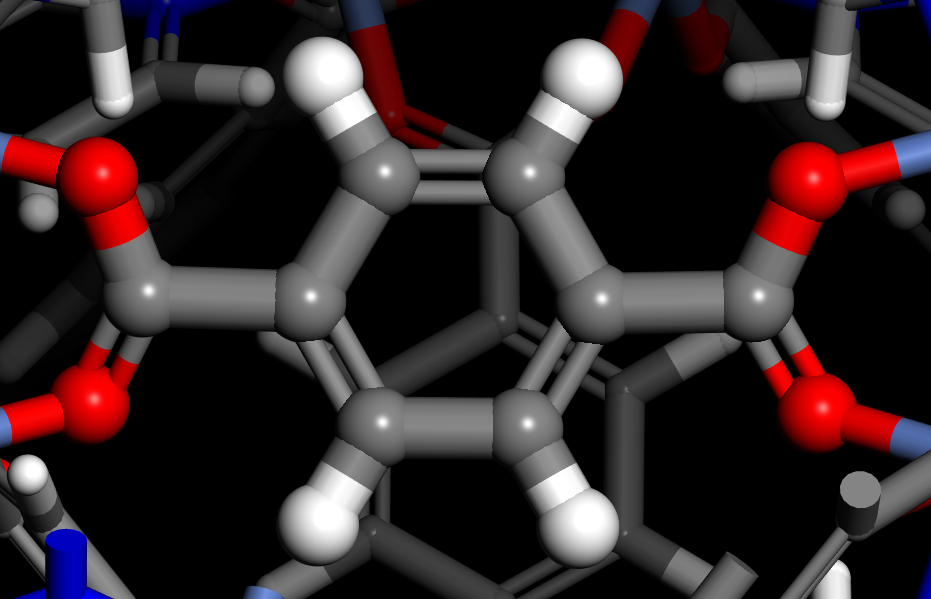

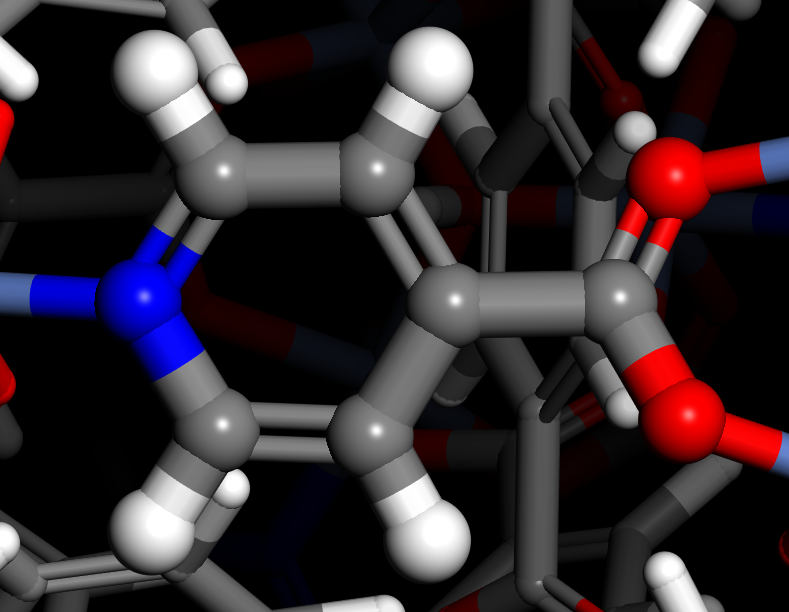


**Supplementary Figure 1.** Two different linkers found in AXUBOL. BDC is shown in the left, and IN is shown in the right.


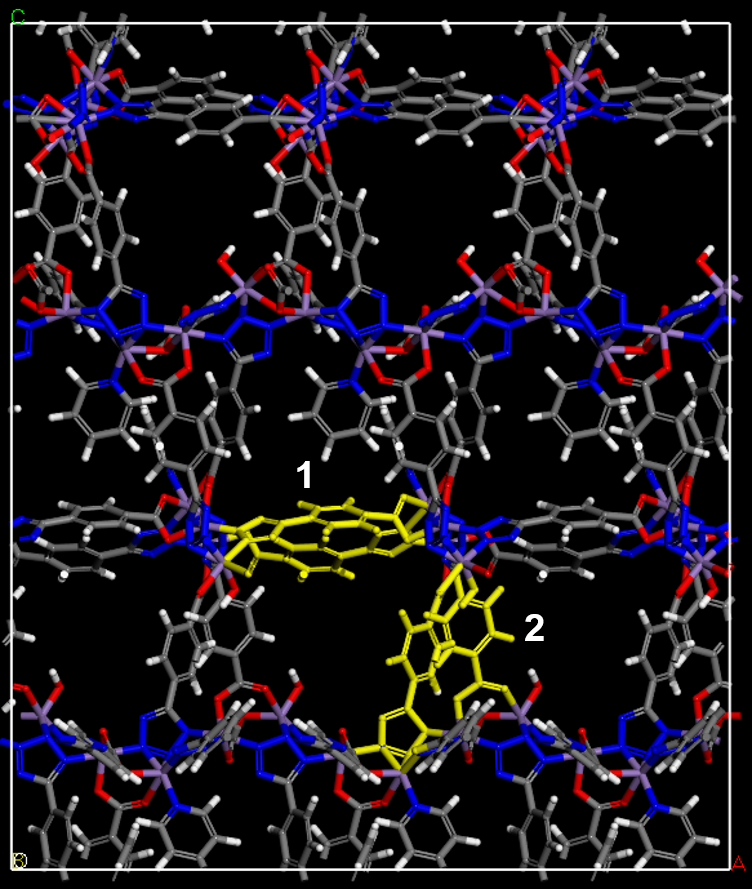


**Supplementary Figure 2.** Two different coordination positions for the same linker were observed in KOCWEF. Indices were assigned for each coordination scenario. Although identical linkers are observed, scheme 1 has the tetrazolate end group bound to two metals, and scheme 2 has tetrazolate end group bound to three metals.

**
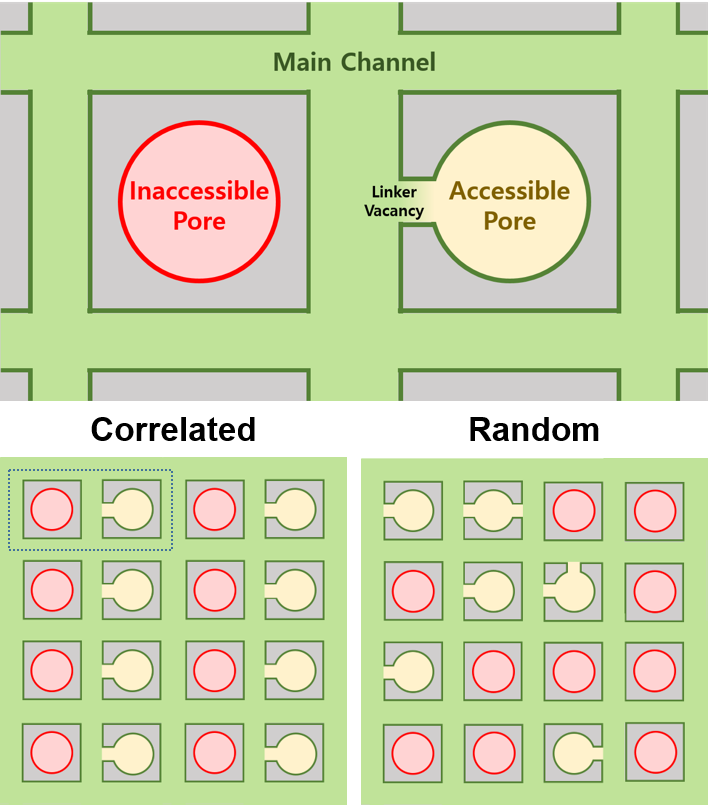
**

**Supplementary Figure 3.** Schematics showing the two different defect distribution scenarios that can be conceived. Note that the proportion of defects within the whole “crystal” remains the same.


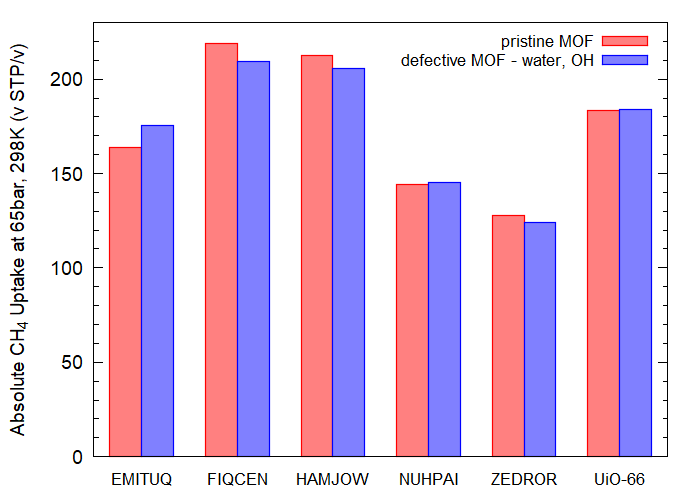


**Supplementary Figure 4.** Methane uptake at 65 bar, 298 K of randomly selected structures from the CoRE MOF database in their pristine and water and hydroxyl coordinated defect form. Clear uptake enhancement trend is not observed for the MOFs selected at random.


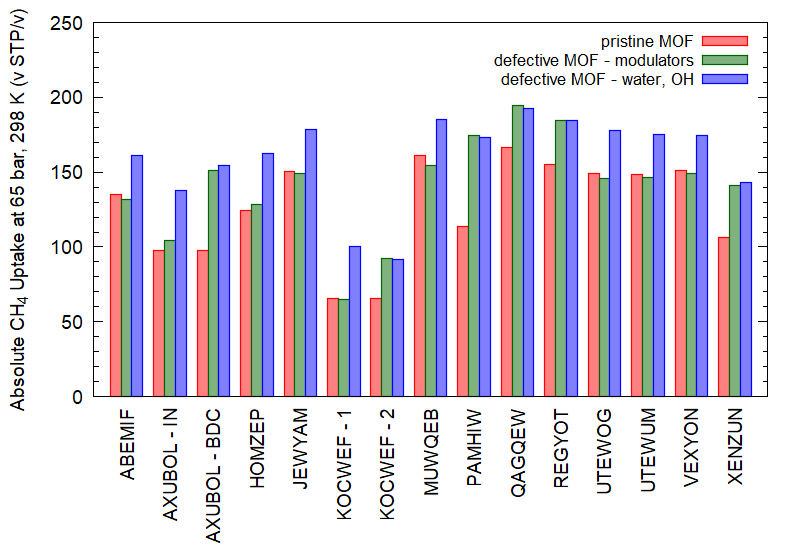


**Supplementary Figure 5.** Methane uptake at 65 bar, 298 K of the candidate MOFs calculated with DREIDING FF. The enhancement trends are well-conserved, meaning that the inaccessibility phenomenon is not dependent on the primarily used force field in our study.


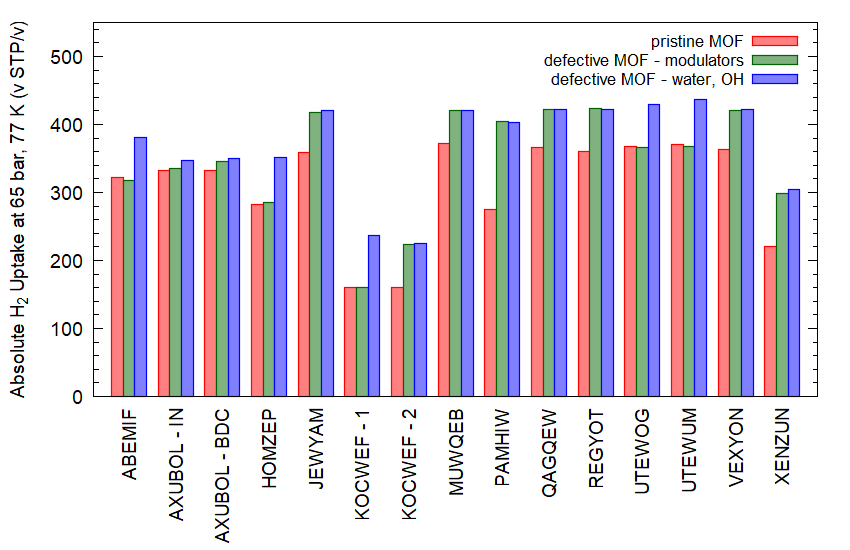


**Supplementary Figure 6.** Hydrogen uptake at 65 bar, 77 K of the candidate MOFs. Most of the enhancement trends observed with methane have carried over to H_2_ gas, meaning that the inaccessibility persists for this smaller guest molecule.


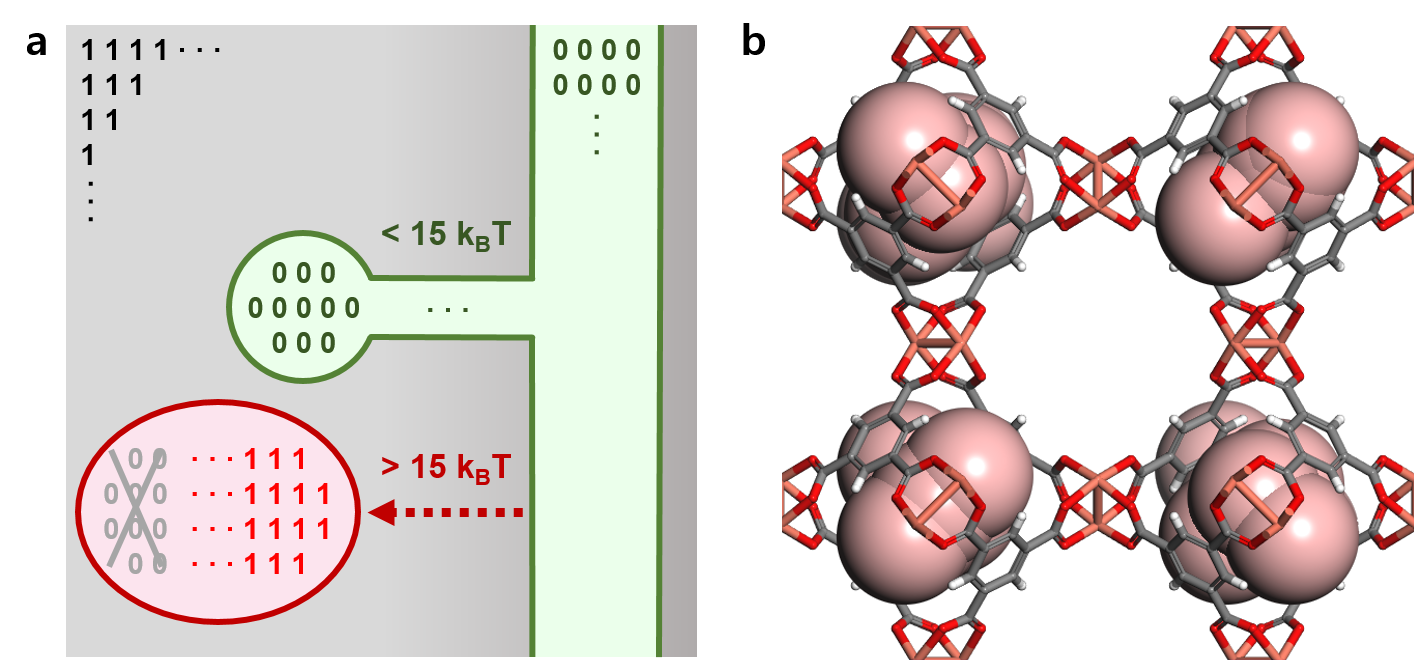


**Supplementary Figure 7. Schematics explaining the blocking algorithm implemented in the GPU code. (a)** A two-dimensional representation of the flood fill algorithm implemented within the GPU code is shown. 0 (green region) denotes the accessible grid points and 1 (grey region) denotes the inaccessible grid points. Secluded pockets of low energy regions, shown in red, are considered to be inaccessible and are effectively blocked with our flood fill algorithm. Only the continuously connected regions of low energy under the energy barrier, shown in green, are considered to be accessible in the calculations. **(b)** Inaccessible regions within HKUST-1, as determined by the GPU code, is presented. The secondary pores (marked by pink spheres) of the framework can be inaccessible towards a large enough adsorbate, and for such adsorbates these pores will not be available for adsorption.


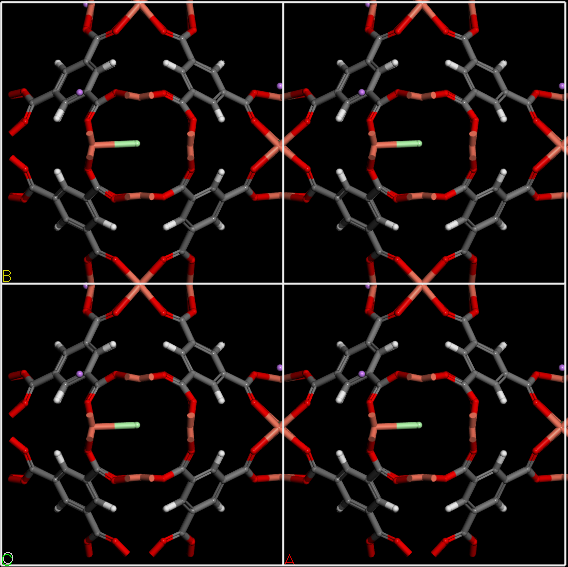


**Supplementary Figure 8.** Structure of ABEMIF visualized in (001) direction. A single unit cell is outlined in white. (Grey: carbon, white: hydrogen, red: oxygen, orange: copper, yellow green: chlorine, purple: lithium)


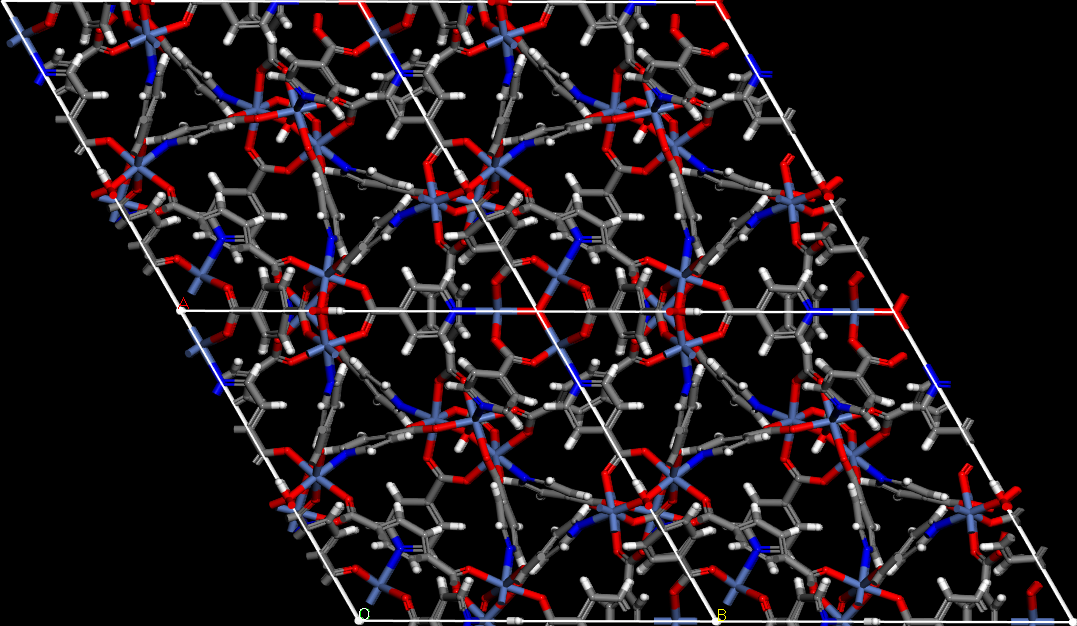


**Supplementary Figure 9.** Structure of AXUBOL visualized in (001) direction. A single unit cell is outlined in white. (Grey: carbon, white: hydrogen, red: oxygen, blue: nitrogen, light blue: nickel)

**
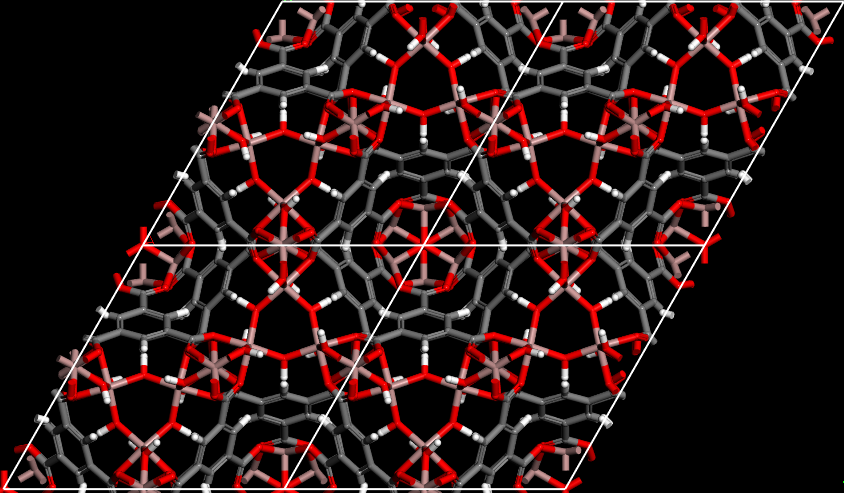
**

**Supplementary Figure 10.** Structure of HOMZEP visualized in (001) direction. A single unit cell is outlined in white. (Grey: carbon, white: hydrogen, brown: gallium)


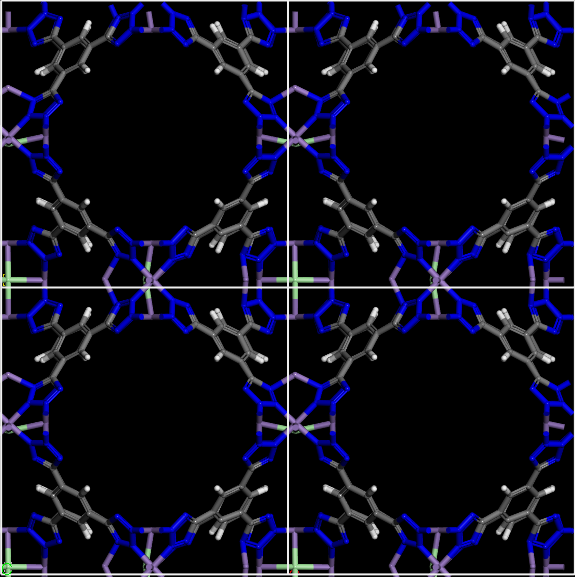


**Supplementary Figure 11.** Structure of JEWYAM visualized in (001) direction. A single unit cell is outlined in white. (Grey: carbon, white: hydrogen, blue: nitrogen, purple: manganese, yellow green: chlorine)


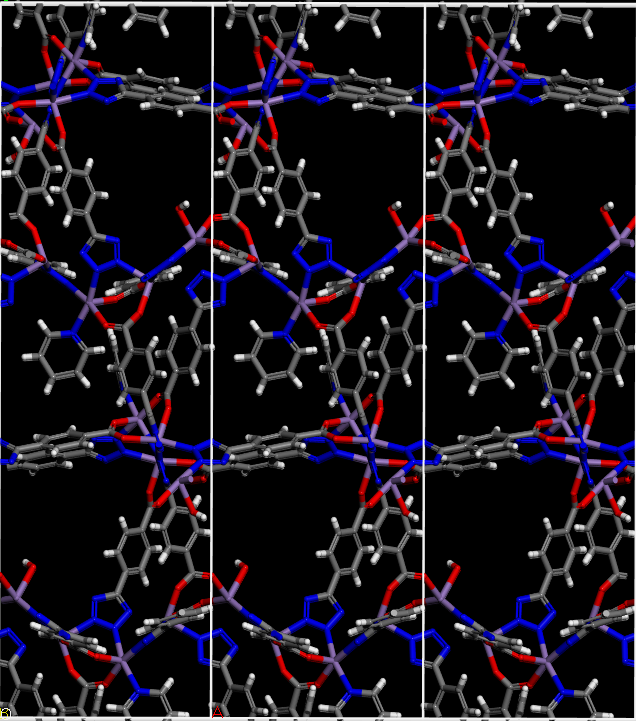


**Supplementary Figure 12.** Structure of KOCWEF visualized in (010) direction. A single unit cell is outlined in white. (Grey: carbon, white: hydrogen, red: oxygen, blue: nitrogen, purple: manganese)


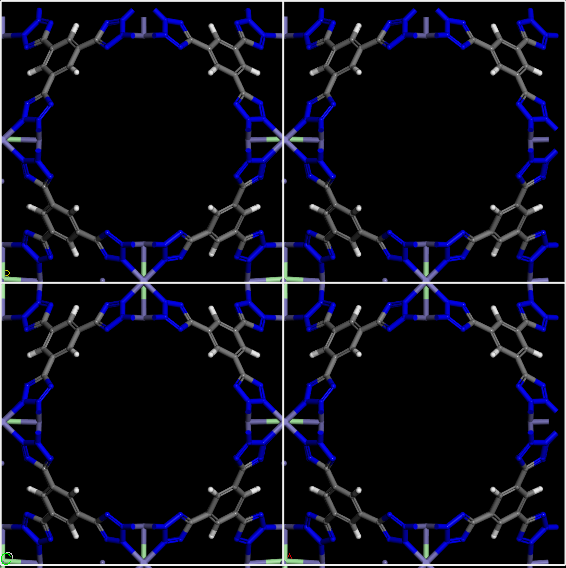


**Supplementary Figure 13.** Structure of MUWQEB visualized in (001) direction. A single unit cell is outlined in white. (Grey: carbon, white: hydrogen, blue: nitrogen, purple: iron, yellow green: chlorine)


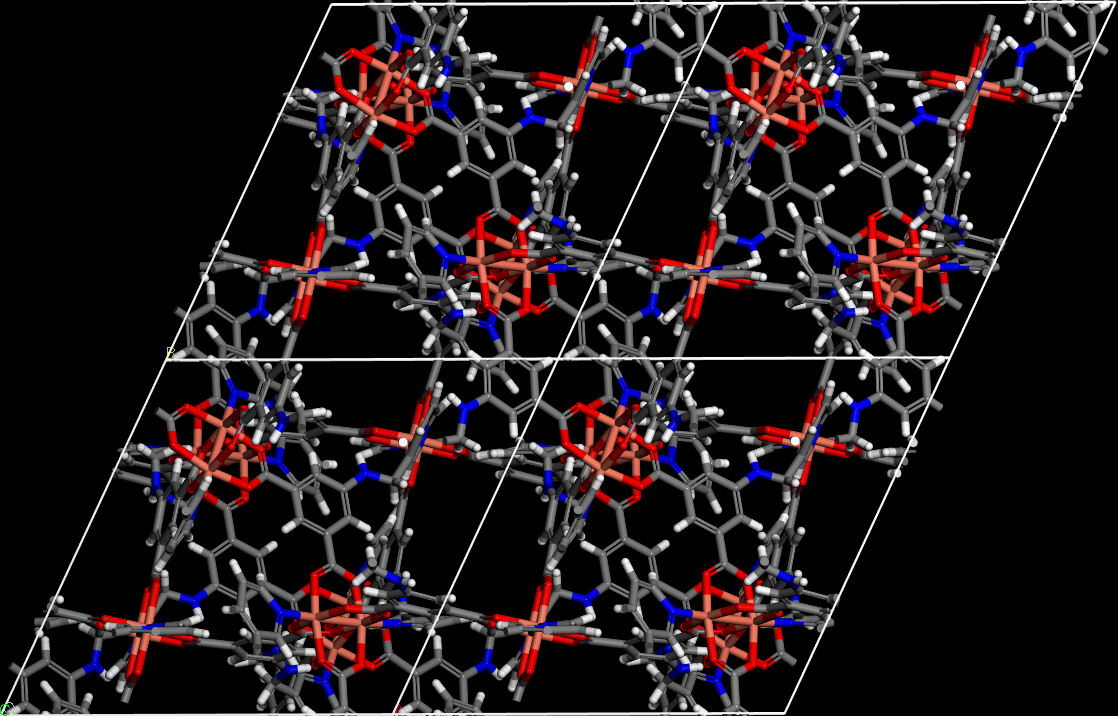


**Supplementary Figure 14.** Structure of PAMHIW visualized in (001) direction. A single unit cell is outlined in white. (Grey: carbon, white: hydrogen, red: oxygen, blue: nitrogen, orange: copper)


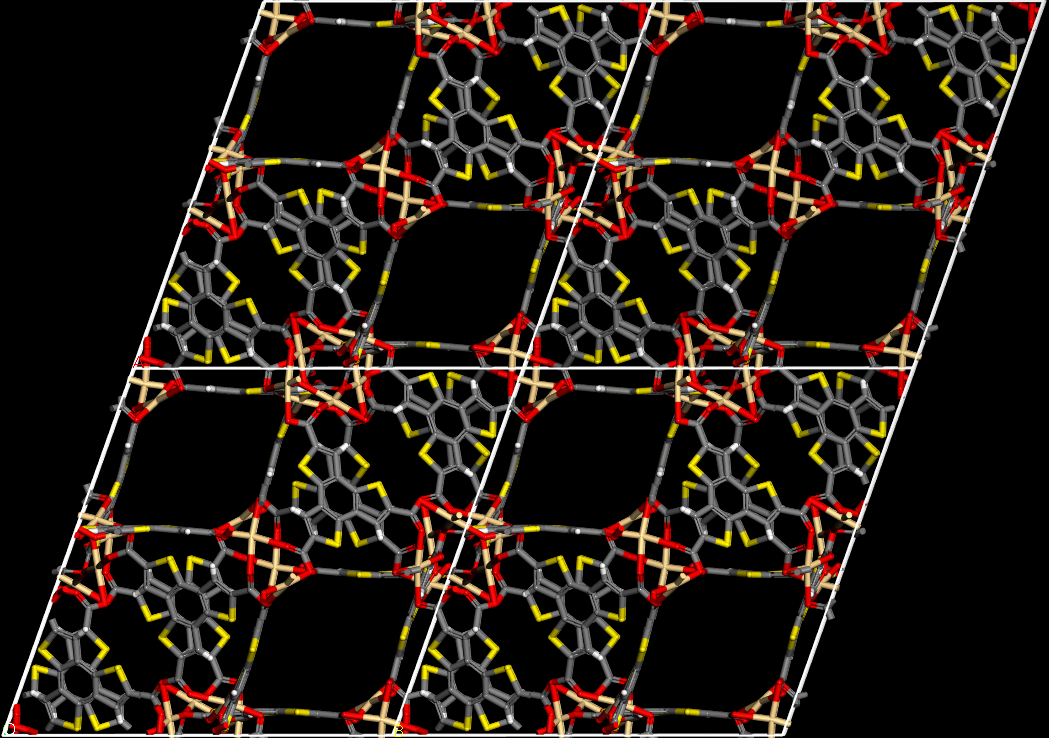


**Supplementary Figure 15.** Structure of QAGQEW visualized in (001) direction. A single unit cell is outlined in white. (Grey: carbon, white: hydrogen, red: oxygen, light yellow: cadmium, yellow: sulfur)


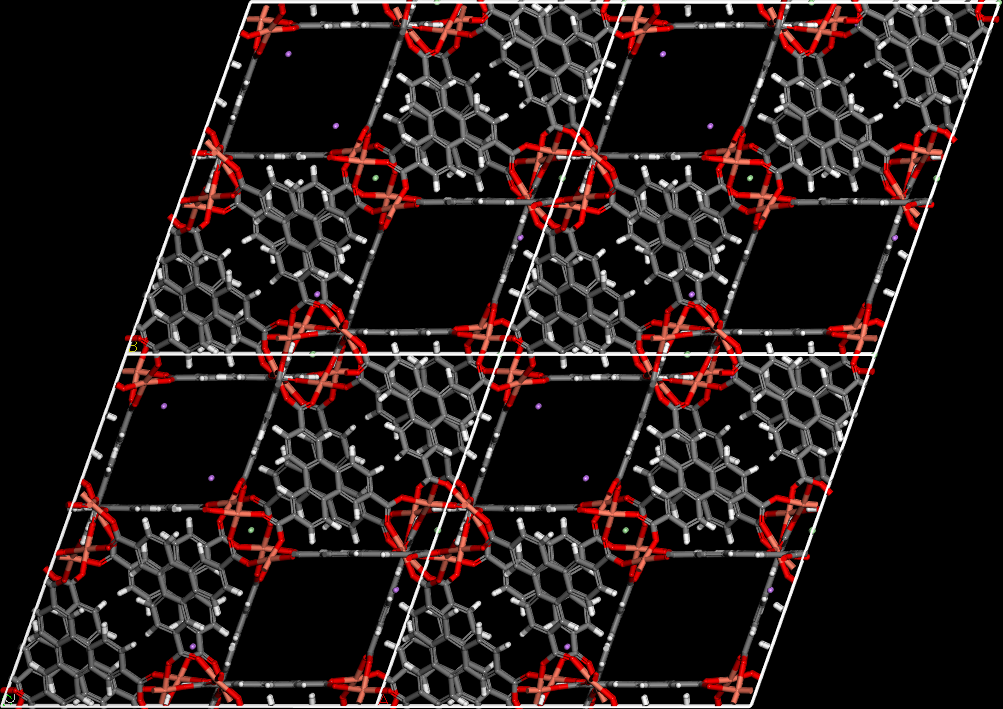


**Supplementary Figure 16.** Structure of REGYOT visualized in (001) direction. A single unit cell is outlined in white. (Grey: carbon, white: hydrogen, red: oxygen, orange: copper, yellow green: chlorine, purple: lithium)


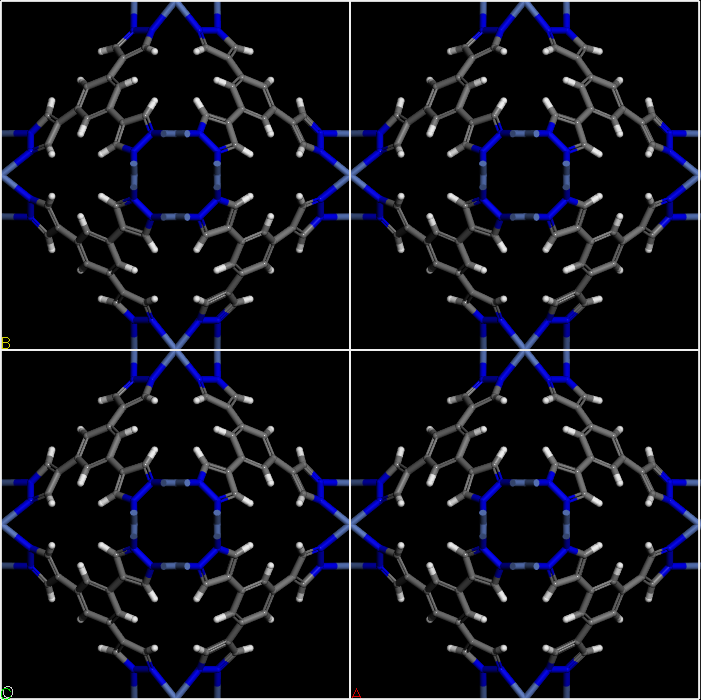


**Supplementary Figure 17.** Structure of UTEWOG visualized in (001) direction. A single unit cell is outlined in white. (Grey: carbon, white: hydrogen, blue: nitrogen, light blue: nickel)


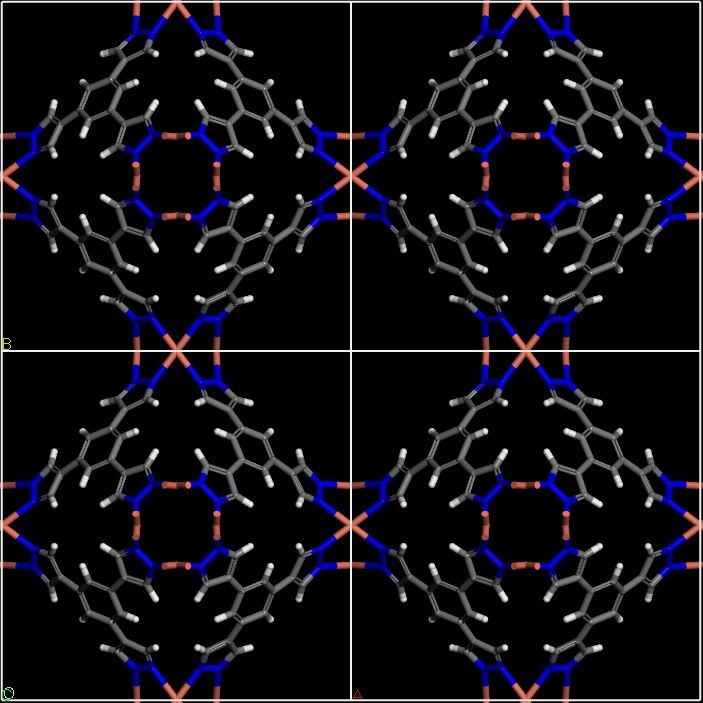


**Supplementary Figure 18.** Structure of UTEWUM visualized in (001) direction. A single unit cell is outlined in white. (Grey: carbon, white: hydrogen, blue: nitrogen, orange: copper)

**
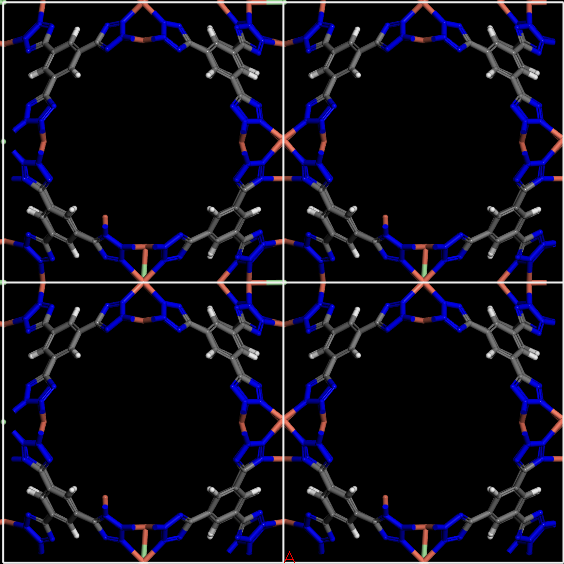
**

**Supplementary Figure 19.** Structure of VEXYON visualized in (001) direction. A single unit cell is outlined in white. (Grey: carbon, white: hydrogen, blue: nitrogen, orange: copper)

**
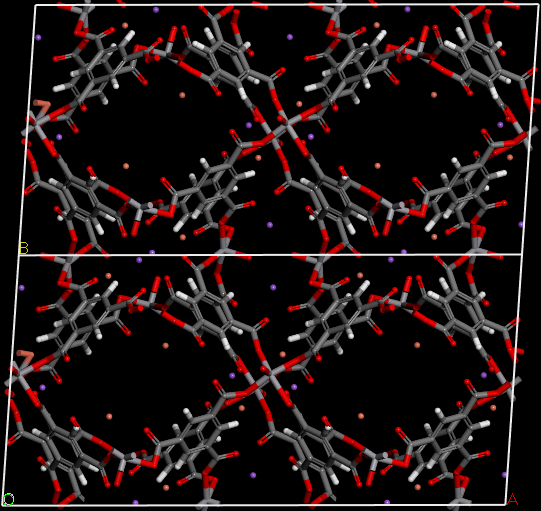
**

**Supplementary Figure 20.** Structure of XENZUN visualized in (001) direction. A single unit cell is outlined in white. (Grey: carbon, white: hydrogen, red: oxygen, silver: vanadium, purple: sodium, orange: copper)


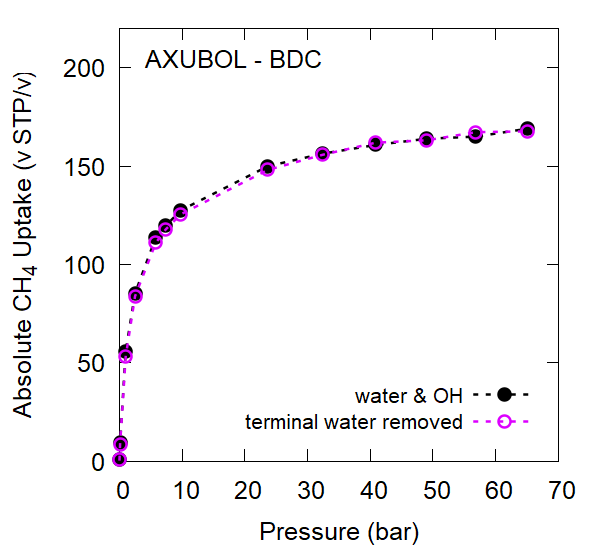

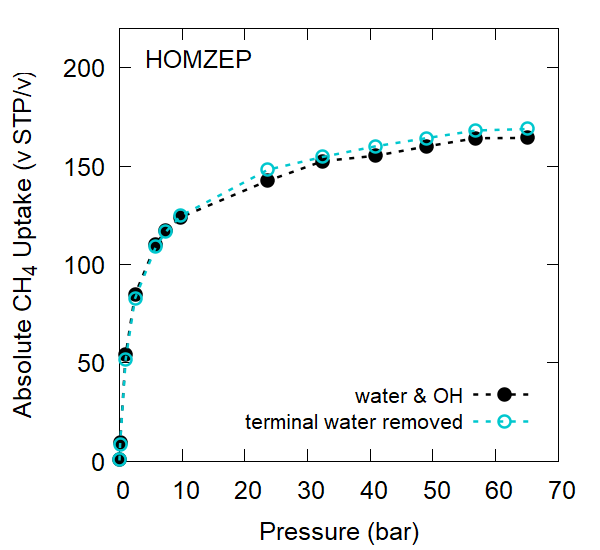


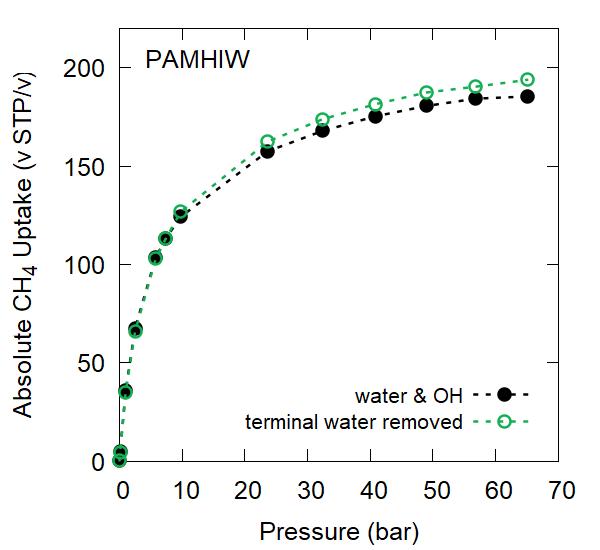

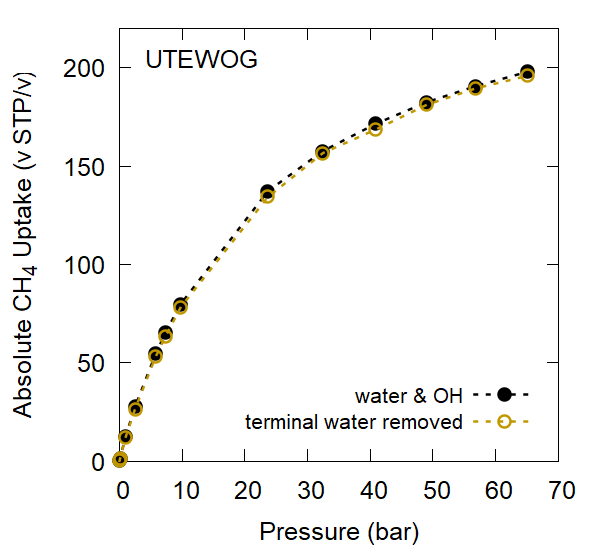


**Supplementary Figure 21.** Sample methane adsorption isotherms at T = 298 K before and after terminal water removal for four candidate MOFs, AXUBOL (BDC linker) – top left, HOMZEP – top right, PAMHIW – bottom left, and UTEWOG – bottom right.


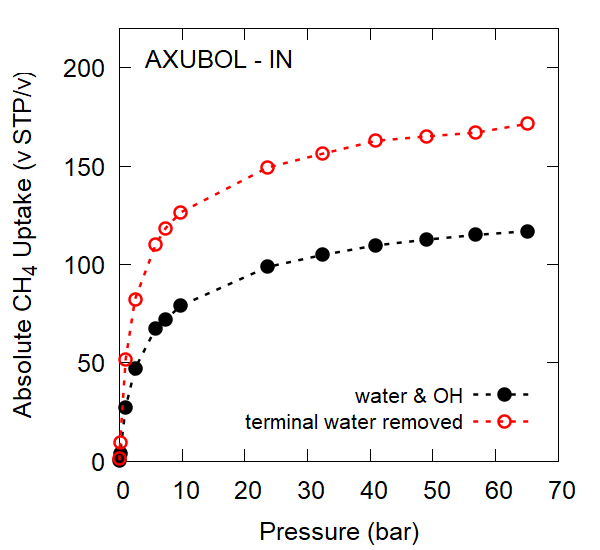

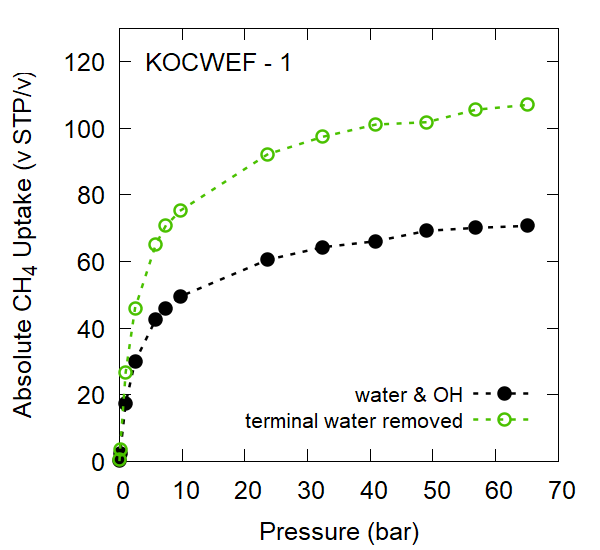


**Supplementary Figure 22.** Change in the methane adsorption isotherms at T = 298 K for IN linker defects in AXUBOL (top) and Linker 1 defects in KOCWEF (bottom) with the removal of terminal water groups.


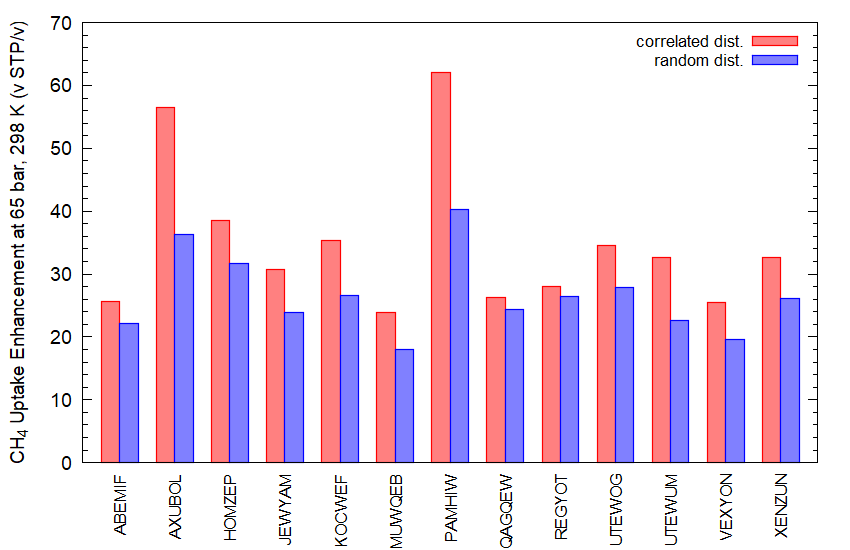


**Supplementary Figure 23.** Methane uptake enhancement at T = 298 K, P = 65 bar for the candidates, under correlated distribution and random distribution of defects within the crystal.


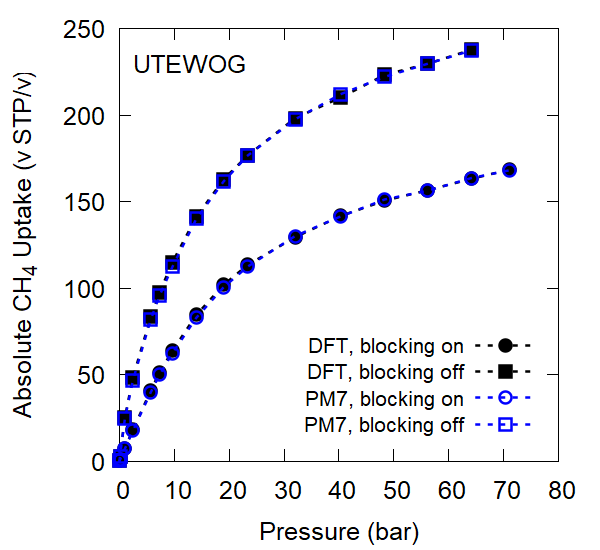

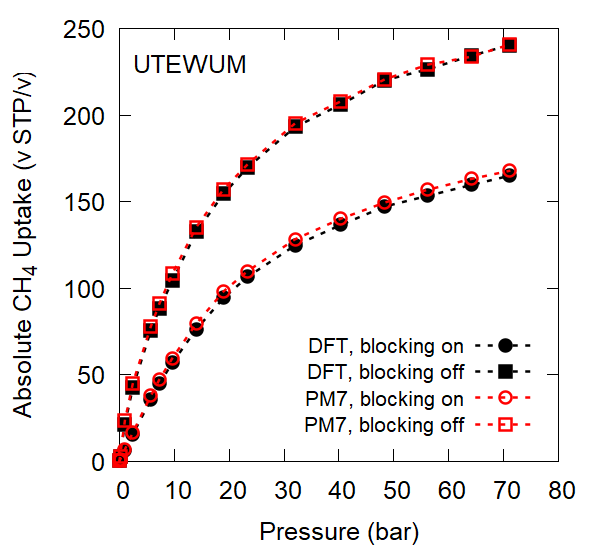


**Supplementary Figure 24.** Methane adsorption isotherms of UTEWOG (top) and UTEWUM (bottom) after being relaxed with semi-empirical PM7 Hamiltonian and DFT.


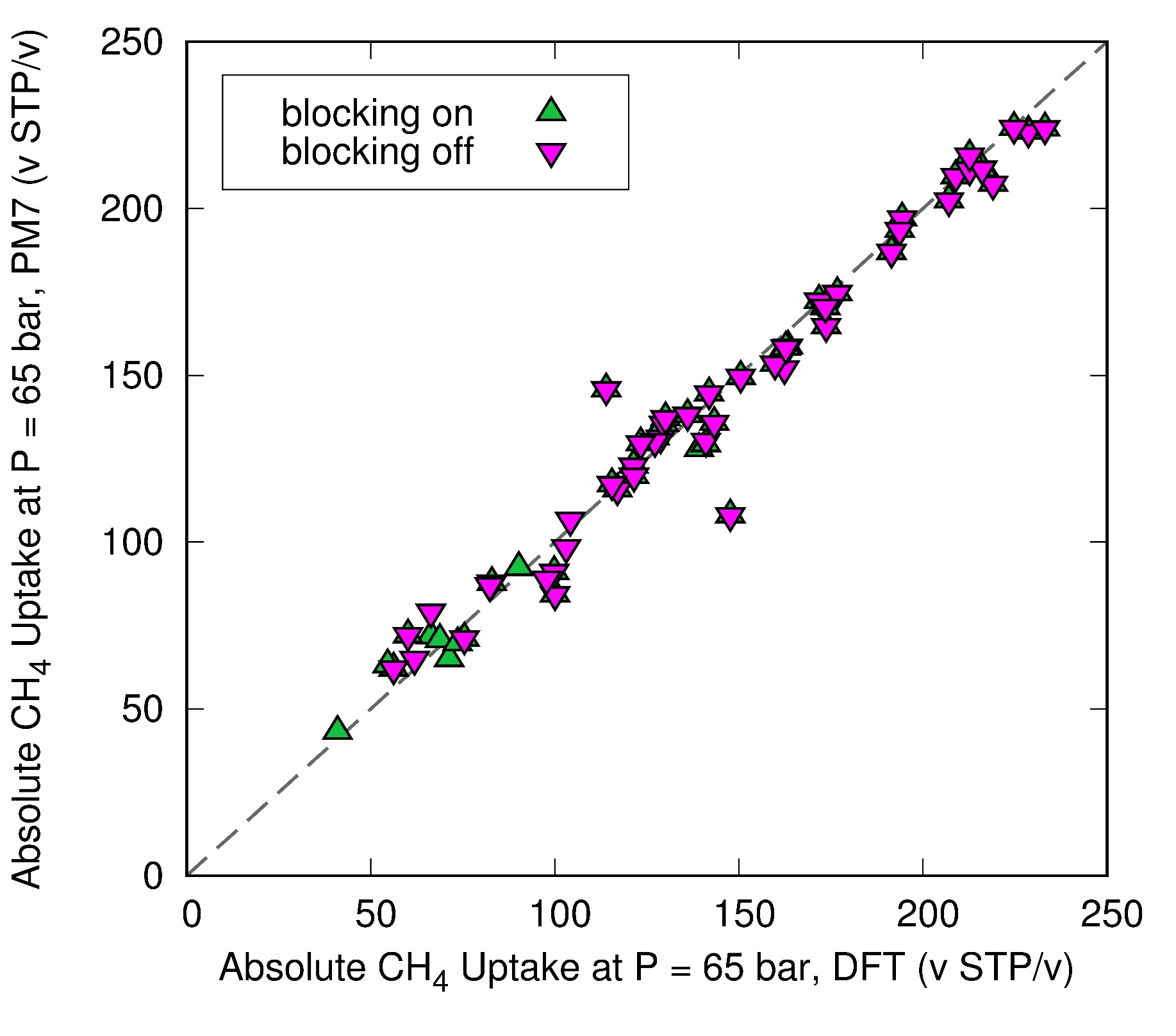


**Supplementary Figure 25.** Comparison of absolute methane uptake at T = 298 K, P = 65 bar for selected MOFs under PM7 and DFT relaxation schemes.

**Supplementary Table 1.** List of 50 additional MOFs (presented by their Cambridge Structure Database reference codes) with significant methane uptake difference between blocking on and off.

| MOF CSD Refcode | Uptake @ 65 bar, blocking off  (v STP/v) | Uptake @ 65 bar, blocking on  (v STP/v) | Uptake difference  (v STP/v) | MOF CSD Refcode | Uptake @ 65 bar, blocking off  (v STP/v) | Uptake @ 65 bar, blocking on  (v STP/v) | Uptake difference  (v STP/v) |
| --- | --- | --- | --- | --- | --- | --- | --- |
| PELWEK | 114.485 | 64.513 | 49.972 | **TEDLUK** | 100.098 | 65.322 | 34.777 |
| HIWKEE | 110.374 | 63.221 | 47.154 | **QIFLIC** | 139.138 | 104.422 | 34.716 |
| ODOXEK | 86.008 | 41.969 | 44.039 | **LEVLEF** | 164.877 | 130.242 | 34.635 |
| YARBUQ | 80.333 | 36.697 | 43.637 | **HUYJUG** | 150.878 | 118.477 | 32.401 |
| DORFUM | 116.831 | 73.218 | 43.613 | **GERWEH** | 211.971 | 179.875 | 32.095 |
| LAGCIH | 131.287 | 87.873 | 43.413 | **BUFPAU** | 177.167 | 145.829 | 31.338 |
| LAGDEE | 146.753 | 103.379 | 43.374 | **XADGAM** | 98.166 | 67.160 | 31.006 |
| GAXWEJ | 92.940 | 50.677 | 42.262 | **BUFNOG** | 177.628 | 147.295 | 30.333 |
| BAHMAZ | 110.379 | 68.153 | 42.226 | **UVUPUX** | 110.023 | 79.777 | 30.246 |
| LAGDOO | 134.698 | 92.800 | 41.898 | **BOXFAW** | 172.738 | 142.601 | 30.137 |
| OHOFEW | 125.875 | 84.510 | 41.365 | **ICANEH** | 131.117 | 101.382 | 29.735 |
| IWELIG | 121.690 | 80.901 | 40.789 | **EYOPUE** | 92.882 | 63.230 | 29.652 |
| WEGDIX | 97.746 | 58.018 | 39.728 | **EYOQAL** | 90.166 | 61.635 | 28.531 |
| IHIWIF | 79.947 | 40.506 | 39.442 | **BUFNEW** | 174.562 | 146.245 | 28.317 |
| LAGDII | 131.198 | 92.096 | 39.102 | **NEXVUI** | 106.583 | 78.730 | 27.853 |
| PENYOX | 72.976 | 34.271 | 38.705 | **GAJTOC** | 68.662 | 41.414 | 27.248 |
| QEFNAQ | 136.640 | 98.000 | 38.639 | **OKIPUU** | 53.488 | 26.460 | 27.028 |
| GULWEQ | 218.033 | 180.204 | 37.829 | **UMUTOM** | 75.362 | 48.434 | 26.928 |
| PEKVAE | 79.601 | 42.695 | 36.906 | **FATKIV** | 134.519 | 107.827 | 26.692 |
| BARZOL | 84.368 | 47.506 | 36.862 | **MIHHAN** | 142.517 | 115.900 | 26.617 |
| QUPJAN | 106.695 | 70.802 | 35.893 | **TETZID** | 97.747 | 71.258 | 26.489 |
| IPICUG | 104.206 | 68.764 | 35.441 | **VOCNOQ** | 60.726 | 34.375 | 26.351 |
| TIMMIM | 67.007 | 31.588 | 35.418 | **BOXFOK** | 197.162 | 171.218 | 25.945 |
| IPIDAN | 102.734 | 67.458 | 35.276 | **HUYKAN** | 148.330 | 122.654 | 25.677 |
| IXURAV | 91.041 | 56.147 | 34.894 | **GAJVAQ** | 66.976 | 42.189 | 24.787 |

**Supplementary Table 2.** List of 32 MOFs with methane uptake difference between blocking on and off of more than 50 (v STP/v), and their coordination environments. Keys for the linker abbreviations within the table is given below.

| MOF CSD Refcode | Uptake @ 65 bar, blocking off  (v STP/v) | Uptake @ 65 bar, blocking on  (v STP/v) | Uptake difference  (v STP/v) | Linker type | linker denticity |
| --- | --- | --- | --- | --- | --- |
| XALCUJ* | 129.711 | 23.632 | 106.079 | XSHZ, BPEA | complicated denticity, 2 |
| XALDIY* | 127.138 | 21.436 | 105.702 | XSHZ, BPEA | complicated denticity, 2 |
| XALDOE* | 130.419 | 25.445 | 104.974 | XSHZ, BPEA | complicated denticity, 2 |
| XAKZAL* | 128.134 | 23.162 | 104.972 | XSHZ, BPEA | complicated denticity, 2 |
| XALDAQ* | 128.402 | 23.951 | 104.451 | XSHZ, BPEA | complicated denticity, 2 |
| XALDEU* | 125.028 | 20.844 | 104.184 | XSHZ, BPEA | complicated denticity, 2 |
| SAKNOJ** | 232.056 | 132.691 | 99.365 | HTDBD | 3 |
| PEYVEV** | 242.189 | 152.950 | 89.239 | BTC | 3 |
| HOMZEP | 238.107 | 152.657 | 85.450 | BTC | 3 |
| EHUFAP** | 124.070 | 41.306 | 82.764 | 1,4-di(1H-imidazol-4-yl)benzene | 2 |
| KOCWEF | 175.552 | 90.357 | 85.195 | 4-TZBA | 2 |
| UTEWOG | 238.196 | 162.791 | 75.405 | BTP | 3 |
| UTEWUM | 234.286 | 160.949 | 73.337 | BTP | 3 |
| DAPBIH* | 186.004 | 118.006 | 67.998 | porphyrin removed, invalid | |
| REGYOT | 234.939 | 167.394 | 67.545 | TTCA | 3 |
| DANZOJ* | 181.197 | 114.194 | 67.003 | porphyrin removed, invalid | |
| XENZUN | 180.059 | 113.264 | 66.795 | BTC | 3 |
| EZOFEF** | 222.920 | 156.864 | 66.057 | BTC | 3 |
| PAMHIW | 194.406 | 129.109 | 65.297 | 5-[(pyridin-3-ylmethynyl)amino]isophthalate | 3 |
| AXUBOL | 198.301 | 134.600 | 63.702 | BDC, IN | 2, 2 |
| OYUJUO* | 202.702 | 138.855 | 63.847 | BDC, IN (same MOF as AXUBOL) | 2, 2 |
| JEWYAM | 194.442 | 132.503 | 61.939 | BTT | 3 |
| XOMJOY** | 138.605 | 77.331 | 61.274 | BDA | 2 |
| ABEMIF | 202.234 | 141.029 | 61.205 | BTC | 3 |
| XOMJUE** | 133.307 | 74.312 | 58.995 | BDA | 2 |
| VEXYON | 190.312 | 132.284 | 58.028 | BTT | 3 |
| VETSUK** | 225.701 | 168.965 | 56.737 | HMTT | 3 |
| QAGQEW | 220.749 | 164.610 | 56.139 | BTTC | 3 |
| MUWQEB | 207.936 | 151.901 | 56.035 | BTT | 3 |
| VETTIZ** | 221.766 | 166.649 | 55.117 | HMTT | 3 |
| VETTAR** | 225.243 | 170.931 | 54.312 | HMTT | 3 |
| VETSOE** | 210.957 | 160.628 | 50.329 | HMTT | 3 |

**Linker Abbreviations**

XSHZ: *N*-acylsalicylhydrazide

BPEA: 1,2-bis(pyridyl)ethane

HTDBD: 4,4’-(6-hydroxy-1,3,5-triazine-2,4-diyl)bis(azanediyl)dibenzoate

BTC: 1,3,5-benzenetricarboxylate

4-TZBA: 4-(1H-tetrazol-5-yl) benzoate

BTP: 1,3,5-benzenetrispyrazolate

TTCA: Triphenylene-2,6,10-tricarboxylate

BDC: 1,4-benzenedicarboxylate

IN: Isonicotinate

BTT: 1,3,5-benzenetristetrazolate

BDA: 6,6A-dichloro-2,2A-diethoxy-1,1A-binaphthalene- 4,4A-dicarboxylate

HMTT: 5,5’,10,10’,15,15’-hexamethyltruxene-2,7,12-tricarboxylate

BTTC: Benzo-(1,2;3,4;5,6)-tris(thiophene-20 -carboxylate)

***:** omitted during structure visualization

****:** omitted due to framework deformation/incompatibility during MOPAC relaxation

**Supplementary Table 3.** List of candidate MOFs and their unit cell volume after semi-empirical relaxation with MOPAC under different defect scenarios.

| MOF CSD Refcode | Pristine volume (Å³) | % linker vacancies | Defect volume – modulators (Å³) | % change in volume | Defect volume - water, OH (Å³) | % change in volume |
| --- | --- | --- | --- | --- | --- | --- |
| ABEMIF | 7714.74 | 6.25 | 7475.31 | 3.10 | 7475.75 | 3.10 |
| AXUBOL  (IN) | 4284.01 | 5.56 | 4282.80 | 0.03 | 4260.33 | 0.55 |
| AXUBOL  (BDC) | 4284.01 | 5.56 | 4269.92 | 0.33 | 4263.03 | 0.49 |
| HOMZEP | 5505.05 | 8.33 | 5503.64 | 0.03 | 5451.06 | 0.98 |
| JEWYAM | 12251.38 | 6.25 | 11934.66 | 2.59 | 11934.62 | 2.59 |
| KOCWEF (1) | 5340.02 | 6.25 | 5348.96 | 0.17 | 5375.01 | 0.66 |
| KOCWEF (2) | 5340.02 | 6.25 | 5341.42 | 0.03 | 5335.84 | 0.08 |
| MUWQEB | 12801.96 | 6.25 | 12568.72 | 1.82 | 12531.15 | 2.16 |
| PAMHIW | 7120.88 | 8.33 | 7138.23 | 0.24 | 7129.18 | 0.12 |
| QAGQEW | 15240.43 | 6.25 | 15273.37 | 0.22 | 15293.63 | 0.35 |
| REGYOT | 17994.07 | 6.25 | 18061.26 | 0.37 | 18035.1 | 0.23 |
| UTEWOG | 13057.26 | 6.25 | 13054.11 | 0.02 | 13043.3 | 0.11 |
| UTEWUM | 13337.32 | 6.25 | 13337.43 | 0.001 | 13361.77 | 0.18 |
| VEXYON | 13026.88 | 6.25 | 12902.88 | 0.95 | 12895.56 | 1.01 |
| XENZUN | 6842.08 | 6.25 | 6654.82 | 2.74 | 6755.64 | 1.26 |

**Supplementary Table 4.** Absolute methane uptake at T = 298 K, P = 65 bar for the final candidate MOFs under different energy thresholds used in detecting inaccessible pores. Uptake is reported in (v STP/v), and is observed to be exactly the same for all candidates under different k_B_T values.

| MOF CSD Refcode | k_B_T = 6 | k_B_T = 9 | k_B_T = 12 | k_B_T = 15 | k_B_T = 18 | k_B_T = 21 | k_B_T = 24 |
| --- | --- | --- | --- | --- | --- | --- | --- |
| ABEMIF | 136.949 | 136.949 | 136.949 | 136.949 | 136.949 | 136.949 | 136.949 |
| AXUBOL | 112.564 | 112.564 | 112.564 | 112.564 | 112.564 | 112.564 | 112.564 |
| HOMZEP | 129.780 | 129.780 | 129.780 | 129.780 | 129.780 | 129.780 | 129.780 |
| JEWYAM | 154.459 | 154.459 | 154.459 | 154.459 | 154.459 | 154.459 | 154.459 |
| KOCWEF | 71.653 | 71.653 | 71.653 | 71.653 | 71.653 | 71.653 | 71.653 |
| MUWQEB | 157.205 | 157.205 | 157.205 | 157.205 | 157.205 | 157.205 | 157.205 |
| PAMHIW | 123.377 | 123.377 | 123.377 | 123.377 | 123.377 | 123.377 | 123.377 |
| QAGQEW | 165.823 | 165.823 | 165.823 | 165.823 | 165.823 | 165.823 | 165.823 |
| REGYOT | 166.004 | 166.004 | 166.004 | 166.004 | 166.004 | 166.004 | 166.004 |
| UTEWOG | 163.434 | 163.434 | 163.434 | 163.434 | 163.434 | 163.434 | 163.434 |
| UTEWUM | 163.191 | 163.191 | 163.191 | 163.191 | 163.191 | 163.191 | 163.191 |
| VEXYON | 152.980 | 152.980 | 152.980 | 152.980 | 152.980 | 152.980 | 152.980 |
| XENZUN | 105.434 | 105.434 | 105.434 | 105.434 | 105.434 | 105.434 | 105.434 |

**Supplementary Table 5.** List of candidate MOFs and their methane uptake enhancement for each defect rate tested in the random defect distribution scheme.

| MOF CSD Refcode | CH_4_ uptake enhancement for each defect rate (v STP/v) | | |
| --- | --- | --- | --- |
|  | **8.33% defects** | **12.50% defects** | **25% defects** |
| ABEMIF | 27.457 | 35.944 | 49.254 |
| AXUBOL | 44.672 | 51.374 | 56.163 |
| HOMZEP | 31.619 | 43.440 | 62.876 |
| JEWYAM | 29.613 | 38.766 | 53.121 |
| KOCWEF | 36.374 | 46.547 | 58.488 |
| MUWQEB | 22.400 | 29.324 | 40.183 |
| PAMHIW | 40.208 | 49.564 | 60.099 |
| QAGQEW | 30.661 | 40.297 | 52.204 |
| REGYOT | 33.476 | 44.206 | 57.571 |
| UTEWOG | 34.711 | 45.440 | 62.266 |
| UTEWUM | 28.119 | 36.811 | 50.442 |
| VEXYON | 24.345 | 31.869 | 43.671 |
| XENZUN | 32.472 | 42.509 | 58.251 |

**Supplementary Table 6.** CSD reference codes of 50 MOFs from the DFT-minimized MOF dataset that were used for the validation of semi-empirical PM7 Hamiltonian. MOFs presented with asterisks had experienced structural deformation during MOPAC calculations.

| ATOXEN | BAEDTA | BUVYEX | CAYDOX | CUHPUR |
| --- | --- | --- | --- | --- |
| DOGZIJ | ECAHAT | ESEVIH | FAPTOH | FEFCUQ |
| FUNCAT | GIFKIP* | GUYLOC | HIFVOI | ICANEH |
| IPICUG | IXOFIL | LAGNUE | LUVTEC | MOCKEV |
| OHAKEO | OHAKIS | PAMVEG | PUWCUF | QEFNAQ |
| QEKLID | QUQFIS | QURSEC | RABHAZ* | RAXCOK |
| RAYLOU | RAZYOI | RURPEA | SUJREV | TETZID |
| UVEXAV | VAGTUU | WALBOC | WIJDAV | XADGAM |
| XEDPIH | XUGSEY | XUVHEB | XUWVEQ | YAZFOW |
| YEGCUJ | YUXQOY | ZASJAG | ZIDDIB | ZOXFAV |

**Supplementary Table 7.** List of MOFs with inaccessible regions after PM7 and DFT relaxation. The uptake difference between blocking and no blocking at P = 65 bar is also given.

| **MOFs with Inaccessible Pores, Sholl DFT** | **Uptake difference with blocking at P = 65 bar (v STP/v)** | **MOFs with Inaccessible Pores, PM7** | **Uptake difference with blocking at P = 65 bar (v STP/v)** |
| --- | --- | --- | --- |
| BAEDTA | 20.877 | BAEDTA | 21.847 |
| ECAHAT | 23.173 | ECAHAT | 24.115 |
| IPICUG | 35.441 | IPICUG | 35.982 |
| QEFNAQ | 36.949 | QEFNAQ | 38.017 |
| TETZID | 26.489 | TETZID | 24.015 |
| XADGAM | 29.461 | XADGAM | 28.800 |
| ZIDDIB | 27.707 | ZIDDIB | 23.884 |
|  |  | **ATOXEN** | 7.376 |
|  |  | **LUVTEC** | 0.983 |

**Supplementary Note 1**

**Chemical Formula and Illustrations of the 13 Final Candidate MOFs**

Here the chemical formula and illustration for each of the 13 final candidate MOFs identified in the study are presented. White silhouette outlines the unit cell of each MOFs as defined in the CoRE MOF database.

**ABEMIF**

Chemical formula (as reported): Li_3_[(Cu_4_Cl)_3_(BTC)_8_]·9DMA

(BTC = 1,3,5-benzenetricarboxylate, DMA = *N*,*N*-dimethylacetamide)

See Supplementary Figure 8.

**AXUBOL**

Chemical formula (as reported): [Ni^II^_2_Ni^III^(μ3-OH)(IN)_3_(BDC)_1.5_]·xSolvent

(BDC = benzene-1,4-dicarboxylate, IN = isonicotinate)

See Supplementary Figure 9.

**HOMZEP**

Chemical formula (as reported): Ga_12_O(OH)_12_({OH}_4_, {H_2_O}_5_)[BTC]_6_ · 24H_2_O

(BTC = 1,3,5-benzenetricarboxylate)

See Supplementary Figure 10.

**JEWYAM**

Chemical formula (as reported): Mn_3_[(Mn_4_Cl)_3_(BTT)_8_(H_2_O)_12_]_2_·42DMF·11H_2_O·20CH_3_OH

(BTT = 1,3,5-benzenetristetrazolate, DMF = *N*,*N*-dimethylformamide)

See Supplementary Figure 11.

**KOCWEF**

Chemical formula (as reported): [Mn_4_(4-TZBA^2-^)_4_(μ2-H_2_O)_2_(H_2_O)(Py)]

(4-TZBA^2-^ = 4-(1H-tetrazol-5-yl) benzoate, Py = pyridine)

See Supplementary Figure 12.

**MUWQEB**

Chemical formula (as reported): Fe_3_[(Fe_4_Cl)_3_(BTT)_8_]_2_·22DMF·32DMSO·11H2O

(BTT = 1,3,5-benzenetristetrazolate, DMF = *N*,*N*-dimethylformamide, DMSO = dimethyl sulfoxide)

See Supplementary Figure 13.

**PAMHIW**

Chemical formula (as reported): [CuL]·*x*DMA·*y*H_2_O

(L = 5-[(Pyridin-3-ylmethyl)amino] isophthalate, DMA = *N*,*N*-dimethylacetamide)

See Supplementary Figure 14.

**QAGQEW**

Chemical formula (as reported): Cd_13_(BTTC)_8_(OH)­_2_(H_2_O)_16_·18DMA

(BTTC = Benzo-(1,2;3,4;5,6)-tris(thiophene-2’-carboxylate), DMA = *N*,*N*-dimethylacetamide)

See Supplementary Figure 15.

**REGYOT**

Chemical formula (as reported): (Li)_3_[(Cu_4_Cl)_3_(TTCA)_8_]·26DEF

(TTCA = triphenylene-2,6,10-tricarboxylate, DEF = *N,N-*diethylformamide)

See Supplementary Figure 16.

**UTEWOG**

Chemical formula (as reported): Ni_3_(BTP)_2_·3DMF·5CH_3_OH·17H2O

(BTP = 1,3,5-tris(pyrazol-4-yl)benzene)

See Supplementary Figure 17.

**UTEWUM**

Chemical formula (as reported): Cu_3_(BTP)_2_·8CH_3_OH·10H_2_O

(BTP = 1,3,5-tris(pyrazol-4-yl)benzene)

See Supplementary Figure 18.

**VEXYON**

Chemical formula (as reported): HCu[(Cu_4_Cl)_3_(btt)_8_]·3.5HCl

(BTT = 1,3,5-benzenetristetrazolate, DMF = *N*,*N*-dimethylformamide)

See Supplementary Figure 19.

**XENZUN**

Chemical formula (as reported): Cu[NaVO(BTC)_4/3_]·0.5DMF

(BTC = 1,3,5-benzenetricarboxylate, DMF = *N*,*N*-dimethylformamide)

See Supplementary Figure 20.

**Supplementary Discussion 1**

**Counter-cation insertion for the anionic MOFs**

It is sometimes the case that the original structure file from the CoRE MOF dataset or Cambridge Structural Database is missing a key component of a given framework. Such was the case for six anionic MOFs, ABEMIF, JEWYAM, MUWQEB, VEXYON, and XENZUN, selected as candidates during the course of material screening in this study. Herein we present the process in which their respective counter-cations were added into the system for correct representation of these frameworks.

All six MOFs, upon consulting the original publication, was found to be able to undergo cation exchange. This then allows for a bulky ion complex or organic cation to be exchanged with metal cations. Such exchange for metal cations was reported for all anionic MOFs in consideration with the exception of ABEMIF. Given that focus of this work is on methane adsorption through all pressure ranges, we thought it was fitting for the anionic MOFs to be coordinated with the smallest cations possible, or the metal cations. This would then guarantee the maximum amount of volume either within the inaccessible pores in the main channels that could be available for CH_4_ uptake.

In the case of JEWYAM, MUWQEB and VEXYON, the experimental positions for metal cations have been reported in the original publication from neutron diffraction results. Then, for these MOFs, appropriate metal cation was located in very close proximity to what can be found in the original literature. Then, additional MOPAC calculations were conducted with the semi empirical PM7 Hamiltonian to stabilize the cations.

In the case of REGYOT, location of the cation was never explicitly stated in the original literature. However, from the fact that a significant difference in high pressure methane uptake between [Et_2_NH_2_]^+^ and Li^+^ was reported, it can be deduced that these metal cations are likely to be found within the main channel. Recognizing that the charge neutralization is mostly needed around the metal cluster, the smaller Lithium cations were located near the metal clusters within the main channel. Then, MOPAC calculations were conducted to stabilize the cations. These calculations turned out to be rather successful, as the resulting structure accurately reproduces the experimental data presented in Figure 7(b), which is the data for REGYOT with Lithium cations.

For ABEMIF, we recognized that the metal cluster type and coordination scheme with carboxylate groups are identical to that of REGYOT. Thus, the exact same procedure with Li^+^ cations was assumed to be also applicable here, and was used in achieving framework neutrality.

As for XENZUN, no information is explicitly given with regards to the exact location of cations. However, the original publication does directly state that the cations are present within the main channels. Thus, a similar approach with REGYOT was used in deducing the metal cation positions within XENZUN using MOPAC.

**Supplementary Discussion 2**

**Terminal water removal in AXUBOL and KOCWEF**

Prior to presenting the special cases of AXUBOL and KOCWEF, we first show that although removal of terminal water groups is a reasonable procedure, it does not have any significant impact on the adsorption trends observed for most of the candidate MOFs. Examples are given in Supplementary Fig. 21 for four candidate MOFs.

It is shown that there exist very little differences in the uptake profiles that arise from the removal of terminal water groups. This can be attributed to several reasons: 1) there are very little terminal water groups to be removed in the first place, since very low defect proportions are being used. 2) there are no significant changes to the amount of interaction that would take place in the MOF with removal of water. 3) the newly created pathway into the inaccessible pore is large enough that the terminal water cannot have an effect on the passage of methane most of the cases. There exist exceptions to this third claim, which are shown in Supplementary Fig. 22 for IN linker defects in AXUBOL, and linker 1 defects in KOCWEF.

For IN linker defects in AXUBOL and linker 1 defects in KOCWEF, even with the expression of defects with the smaller coordination groups of water and OH, resulting linker vacant site was not significant enough to allow for methane to diffuse into the inaccessible pores under the blocking condition used. Hence, a significant increase in methane uptake can be observed with the removal of residual water groups, as only then will the pathway become large enough for methane to pass into the inaccessible pores. This is shown in above graphs.

Removal of terminal water groups does not disturb the essential coordination environment at the metal clusters. In fact, with a proper activation of the framework under experimental settings, terminal water groups can easily evaporate away from the framework. From the list of defect scenarios, only the IN linker defect case for AXUBOL and linker 1 defect case for KOCWEF were showing no uptake enhancement can be achieved initially with the full expression of coordinating groups. Hence, for these cases only, terminal water groups are removed as a rationalized secondary measure to further test for any uptake enhancements that fundamentally arise from linker vacancies.

**Supplementary Discussion 3**

**Results under the random defect distribution scenario**

In studying the different distributions of linker defects, this study considers two different cases: correlated and random. In a correlated distribution, a single defect is introduced to the unit cell, and then this defect configuration is then replicated infinitely in all three dimensions. This results in equal volume of inaccessible pores being opened up per each cell. In a random distribution, the number of defects varies randomly, and there is no longer any kind of order in the defect configurations. This amounts to some unit cells with no linker defects and thus no enhancement, and some unit cells with multiple linker defects. These defect distribution scenarios are shown in Supplementary Fig. 3. Because it is only the correlated distribution of defects that can be directly dealt with in the GCMC simulation scheme being used, results for the correlated scenario has been presented in the main text. The case of random defect distribution can still be studied, however, with a linear combination approximation of unit cells with different number of defects.

In considering the random defect distribution case, it was assumed that the overall adsorption property of the defective MOF crystal can be described by a linear combination between unit cells with different defect proportions. By utilizing a simple code that randomly generates the number of defects per unit cell for 1,000,000 different unit cells in total, we obtained the number of unit cells with different number of defects ranging from 0, 1, 2, … to the total number of linkers present in the unit cell. The unit cells were taken to be the most primitive unit cell of the given MOF, in which having a single defect already opens up all of the inaccessible volume that is present in most cases. This meant that the pristine cells can be assumed to have the adsorption properties of a pristine MOF, and any of the defect cells can be assumed to have the adsorption properties from of a defective MOF with the maximum uptake enhancement. In a few cases where there exist two secluded inaccessible pores (HOMZEP, KOCWEF, PAMHIW) in the pristine unit cell, we made the assumption that the second defect would be as far as possible from the first defect, and thus it would always open up the second inaccessible pore. This assumption was made with the concern that neighboring defects would complicate the overall stability of the MOF structure. For these MOFs, pristine unit cells would have the pristine MOF adsorption properties, single defect unit cells would exhibit adsorption behavior calculated for a unit cell with only one inaccessible pore affected, and only the multiple defect unit cells would have the adsorption properties with maximum uptake enhancement. The adsorption property of each defect scenario is obtained from a sample GCMC simulation having corresponding number of inaccessible pores open.

Then, using the resulting proportion of unit cells with different number of defects, weight applied to each enhancement scenario was determined, and a simple linear combination was performed to approximate the resulting adsorption profile of the defect crystal with random distribution of defects. The results at P = 65 bar for each of the candidate MOFs is shown in Supplementary Fig. 23 and compared with the correlated defect scenario. The total proportion of linker defects in the crystal used in calculating the random defect distribution scenario was matched with what was used in the correlated scheme for each candidate MOF. Note that these values can be different between different MOFs, and the defect rates are presented in Table 1.

With the exception of AXUBOL and PAMHIW, the difference between correlated and random distribution is observed to be minimal. This is because each of the candidate MOFs contained multiple secluded inaccessible pores within the unit cell that was initially used for defect introduction. Hence, there still existed significant number of inaccessible pores in the crystal even after the introduction of defects. AXUBOL and PAMHIW, on the other hand, were cases where all of the inaccessible pore within the unit cell that was being opened up with a single defect introduction. Thus, random assumption significantly lowers the predicted uptake enhancement.

The use of the linear combination approximation makes it easy to test several other defect rates for the candidate MOFs, even beyond the arbitrary limit of 8.333% used in this study, which cannot be easily tested with explicit GCMC simulations. The uptake enhancement calculated for each candidate MOF under several other linker defect rates, now all equal for each MOF, are presented in Supplementary Table 5. With increasing proportion of defects, there also comes a significant increase in the uptake enhancement that can be achieved. However, it must be noted that such high linker defect rates can comprise the stability of the framework.

**Supplementary Discussion 4**

**Validation of semi-empirical PM7 Hamiltonian and MOPAC**

In accurately predicting the gas adsorption properties of a metal-organic framework, energy minimization or structure relaxation becomes a very important task. It is very often the case that original framework coordinates, as found from the CoRE MOF dataset or the Cambridge Structural Database, are representative of the framework that contains solvents. However, for gas adsorption, these materials are put through an activation process that releases these solvents and maximizes the void volume. Then, with the removal of solvents, slight relocations of the framework atoms to find a new energy minimum becomes inevitable. As such, it is very important for one to perform energy minimization on the unrelaxed MOF structures.

Energy minimization can be performed via several different methods, ranging from ab initio to semi-empirical and classical methods. Although energy minimization using DFT would be the most ideal for using the highest theory among three, it requires a tremendous amount of time and computing resource to conduct DFT energy minimizations for all candidates and their defect derivatives. Thus, to complete the energy minimization task with reasonable accuracy and time-efficiency, this study primarily utilized the semi-empirical methods using the PM7 Hamiltonian with MOPAC software. Use of semi-empirical methods on MOFs, though validated in a few previous publications (DOI: 10.1021/acs.jpcc.5b05599, DOI: 10.1021/jp401920y), can raise concerns since its accuracy cannot be guaranteed for MOF systems. Thus, it is necessary for one to validate the use of such semi-empirical methods, at least within the scope of the research being conducted. As such, herein we validate the use of PM7 against DFT for the prediction of methane uptake and detection of inaccessible pores in MOFs porous towards methane.

In validating the semi-empirical MOPAC calculations with PM7 Hamiltonian, the DFT-minimized MOF structures from Nazarian and Sholl et al. (DOI: 10.1021/acs.chemmater.6b04226) were used for comparison. Methane GCMC simulations were conducted on the final configurations of PM7 and DFT relaxation schemes, and the results were compared. Among the 13 final candidates presented in this study, UTEWOG and UTEWUM were found in the DFT-minimized structure database. Shown above in Supplementary Fig. 24, the GCMC results of these MOFs show almost perfect agreement between the two relaxation schemes. Also, the flood fill algorithm also detects the inaccessible regions consistently under both relaxation schemes.

Validation work with only two MOFs is far too small of a sample size, and thus we expanded the procedure to 50 additional MOFs randomly chosen from the DFT minimized MOF dataset. 6 MOFs with (K_H_, blocking off)/(K_H_, blocking on) > 1.5 as identified during the screening process were first chosen. In the process of selecting the remaining 44 frameworks, Zeo++ (DOI: 10.1016/j.micromeso.2011.08.020) was used to calculate the largest free diameter of a given framework. Only the frameworks with largest free diameter of higher than 3.5 Å were considered to guarantee porosity towards methane, which will set the grounds for a fair evaluation of the blocking phenomenon under different relaxation schemes. Supplementary Table 6 shows the list of MOFs that were selected for PM7 validation. Each of the selected MOF was relaxed using MOPAC with PM7. Visualization of the final configurations from MOPAC relaxation revealed that two of the selected MOFs, GIFKIP and RABHAZ, underwent an unwanted linker detachment around the metal clusters. Following suit of the actual screening process, these MOFs were omitted from further validation process.

On the remaining 48 MOFs, methane GCMC calculations were conducted on both the PM7-relaxed and DFT-relaxed structures at T = 298 K for comparison. The calculations were conducted twice, once with blocking and once without blocking. The results at P = 65 bar is presented in Supplementary Fig. 25. In general, there exists good agreement for each MOF between its PM7 and DFT relaxed configurations, as most of the data points fall along the y = x line in grey. There are a few outliers however, such as WALBOC or YEGCUJ that shows an absolute uptake difference of 39.578 (v STP/v) and 31.820 (v STP/v) respectively between the two relaxation schemes. Overall, the agreement between two relaxation schemes is great, with an R^2^ value of 0.963 when fitted to y = x.

In addition to analyzing the uptake difference, the blocking phenomenon carryover between the two relaxation schemes was also studied. Supplementary Table 7 shows the list of MOFs with inaccessible pores as detected by the flool-fill algorithm after each relaxation method was used on the same MOF structure. First seven structures from both relaxation schemes are identical, meaning that PM7 relaxation performs well enough for the blocking trend observed from DFT relaxation to be retained. What meay seem concerning is the additional inclusion of ATOXEN and LUVTEC under PM7 relaxation, which does not contain any inaccessible pores under DFT relaxation. However, it must be noted that the uptake difference at P = 65 bar between blocking on and off is a mere 7.376 (v STP/v) for ATOXEN, and 0.983 (v STP/v) for LUVTEC. These are residual amounts of inaccessible regions that are too small in volume, and would be filtered out during the course of our screening process. Hence, PM7 can correctly predict all seven MOFs with significant volume of inaccesible regions, as found from the DFT relaxed configurations.

Through the validation process, it is shown that MOPAC energy minimization with semi-empirical PM7 Hamiltonian is an acceptable method for structure relaxation within the context of this study. 50 structures with similar porosity towards methane with the candidate MOFs show good agreement in GCMC calculation and blocked pore detection results between the two relaxation schemes. The inaccuracies of PM7 does exist, as shown by the omission of deformed structures, outliers with signifcant methane uptake difference, and detection of new MOFs with residual inaccesible volume. These inaccuracies cannot hinder the screening process, however, as the MOFs showing such discrepancies would be effectively filtered from the candidate list with the screening criteria implemented.
