# Supplementary material for: Excavating hidden adsorption sites in metal-organic frameworks using rational defect engineering
Source: Nat Commun. 2017 Nov 16;8:1539. doi: 10.1038/s41467-017-01478-4 (PMC5691151; doi:10.1038/s41467-017-01478-4)
Supplement: Supplementary file 3 — Description of Additional Supplementary Files [file 41467_2017_1478_MOESM3_ESM.pdf]

Description of Additional Supplementary Files

File Name: Supplementary Data 1
Description: Crystallographic Information File that contains the relaxed configuration of pristine ABEMIF.

File Name: Supplementary Data 2
Description: Crystallographic Information File that contains the relaxed configuration of defective ABEMIF with modulators.

File Name: Supplementary Data 3
Description: Crystallographic Information File that contains the relaxed configuration of defective ABEMIF with water & OH.

File Name: Supplementary Data 4
Description: Crystallographic Information File that contains the relaxed configuration of pristine AXUBOL.

File Name: Supplementary Data 5
Description: Crystallographic Information File that contains the relaxed configuration of defective AXUBOL with modulators replacing BDC linker.

File Name: Supplementary Data 6
Description: Crystallographic Information File that contains the relaxed configuration of defective AXUBOL with water & OH replacing BDC linker.

File Name: Supplementary Data 7
Description: Crystallographic Information File that contains the relaxed configuration of defective AXUBOL with modulators replacing IN linker.

File Name: Supplementary Data 8
Description: Crystallographic Information File that contains the relaxed configuration of defective AXUBOL with water & OH replacing IN linker.

File Name: Supplementary Data 9
Description: Crystallographic Information File that contains the relaxed configuration of pristine HOMZEP.

File Name: Supplementary Data 10
Description: Crystallographic Information File that contains the relaxed configuration of defective HOMZEP with modulators.

File Name: Supplementary Data 11
Description: Crystallographic Information File that contains the relaxed configuration of defective HOMZEP with water & OH.

File Name: Supplementary Data 12

Description: Crystallographic Information File that contains the relaxed configuration of pristine JEWYAM.

File Name: Supplementary Data 13
Description: Crystallographic Information File that contains the relaxed configuration of defective JEWYAM with modulators.

File Name: Supplementary Data 14
Description: Crystallographic Information File that contains the relaxed configuration of defective JEWYAM with water & OH.

File Name: Supplementary Data 15
Description: Crystallographic Information File that contains the relaxed configuration of pristine KOCWEF.

File Name: Supplementary Data 16
Description: Crystallographic Information File that contains the relaxed configuration of defective KOCWEF with modulators replacing Linker 1.

File Name: Supplementary Data 17
Description: Crystallographic Information File that contains the relaxed configuration of defective KOCWEF with water & OH replacing Linker 1.

File Name: Supplementary Data 18
Description: Crystallographic Information File that contains the relaxed configuration of defective KOCWEF with modulators replacing Linker 2.

File Name: Supplementary Data 19
Description: Crystallographic Information File that contains the relaxed configuration of defective KOCWEF with water & OH replacing Linker 2.

File Name: Supplementary Data 20
Description: Crystallographic Information File that contains the relaxed configuration of pristine MUWQEB.

File Name: Supplementary Data 21
Description: Crystallographic Information File that contains the relaxed configuration of defective MUWQEB with modulators.

File Name: Supplementary Data 22
Description: Crystallographic Information File that contains the relaxed configuration of defective MUWQEB with water & OH.

File Name: Supplementary Data 23
Description: Crystallographic Information File that contains the relaxed configuration of pristine PAMHIW.

File Name: Supplementary Data 24
Description: Crystallographic Information File that contains the relaxed configuration of defective PAMHIW with modulators.

File Name: Supplementary Data 25
Description: Crystallographic Information File that contains the relaxed configuration of defective PAMHIW with water & OH.

File Name: Supplementary Data 26
Description: Crystallographic Information File that contains the relaxed configuration of pristine QAGQEW.

File Name: Supplementary Data 27
Description: Crystallographic Information File that contains the relaxed configuration of defective QAGQEW with modulators.

File Name: Supplementary Data 28
Description: Crystallographic Information File that contains the relaxed configuration of defective QAGQEW with water & OH.

File Name: Supplementary Data 29
Description: Crystallographic Information File that contains the relaxed configuration of pristine REGYOT.

File Name: Supplementary Data 30
Description: Crystallographic Information File that contains the relaxed configuration of defective REGYOT with modulators.

File Name: Supplementary Data 31
Description: Crystallographic Information File that contains the relaxed configuration of defective REGYOT with water & OH.

File Name: Supplementary Data 32
Description: Crystallographic Information File that contains the relaxed configuration of pristine UTEWOG.

File Name: Supplementary Data 33
Description: Crystallographic Information File that contains the relaxed configuration of defective UTEWOG with modulators.

File Name: Supplementary Data 34
Description: Crystallographic Information File that contains the relaxed configuration of defective UTEWOG with water & OH.

File Name: Supplementary Data 35
Description: Crystallographic Information File that contains the relaxed configuration of pristine UTEWUM.

File Name: Supplementary Data 36
Description: Crystallographic Information File that contains the relaxed configuration of defective UTEWUM with modulators.

File Name: Supplementary Data 37
Description: Crystallographic Information File that contains the relaxed configuration of defective UTEWUM with water & OH.

File Name: Supplementary Data 38
Description: Crystallographic Information File that contains the relaxed configuration of pristine VEXYON.

File Name: Supplementary Data 39
Description: Crystallographic Information File that contains the relaxed configuration of defective VEXYON with modulators.

File Name: Supplementary Data 40
Description: Crystallographic Information File that contains the relaxed configuration of defective VEXYON with water & OH.

File Name: Supplementary Data 41
Description: Crystallographic Information File that contains the relaxed configuration of pristine XENZUN.

File Name: Supplementary Data 42
Description: Crystallographic Information File that contains the relaxed configuration of defective XENZUN with modulators.

File Name: Supplementary Data 43
Description: Crystallographic Information File that contains the relaxed configuration of defective XENZUN with water & OH.
